# Supplementary material for: COZOID: contact zone identifier for visual analysis of protein-protein interactions
Source: BMC Bioinformatics. 2018 Apr 6;19:125. doi: 10.1186/s12859-018-2113-6 (PMC5889581; doi:10.1186/s12859-018-2113-6)
Supplement: Supplementary file 2 — Software build. Executable binary file of the software tool. (ZIP 118,784 kb) [file 12859_2018_2113_MOESM2_ESM.zip › cozoid/java/docs/beansbinding-1.2.1-doc.zip!/constant-values.html]

Constant Field Values (Beans Binding)


---


|  |  |  |
| --- | --- | --- |
| **Overview** \| Package \| Class \| Use \| **Tree** \| **Deprecated** \| **Index** \| **Help** | |  |
| PREV   NEXT | **FRAMES**    **NO FRAMES**     **All Classes** |  |


---


# Constant Field Values


---

**Contents**

- org.jdesktop.\*

org.jdesktop.\*

| org.jdesktop.el.ELResolver | | |
| --- | --- | --- |
| `public static final java.lang.String` | `RESOLVABLE_AT_DESIGN_TIME` | `"resolvableAtDesignTime"` |
| `public static final java.lang.String` | `TYPE` | `"type"` |

| org.jdesktop.el.impl.parser.ELParserConstants | | |
| --- | --- | --- |
| `public static final int` | `AND0` | `39` |
| `public static final int` | `AND1` | `40` |
| `public static final int` | `BADLY_ESCAPED_STRING_LITERAL` | `13` |
| `public static final int` | `COLON` | `23` |
| `public static final int` | `COMMA` | `24` |
| `public static final int` | `DEFAULT` | `0` |
| `public static final int` | `DIGIT` | `57` |
| `public static final int` | `DIV0` | `49` |
| `public static final int` | `DIV1` | `50` |
| `public static final int` | `DOT` | `18` |
| `public static final int` | `EMPTY` | `43` |
| `public static final int` | `END_EXPRESSION` | `17` |
| `public static final int` | `EOF` | `0` |
| `public static final int` | `EQ0` | `33` |
| `public static final int` | `EQ1` | `34` |
| `public static final int` | `EXPONENT` | `11` |
| `public static final int` | `FALSE` | `15` |
| `public static final int` | `FLOATING_POINT_LITERAL` | `10` |
| `public static final int` | `FUNCTIONSUFFIX` | `54` |
| `public static final int` | `GE0` | `29` |
| `public static final int` | `GE1` | `30` |
| `public static final int` | `GT0` | `25` |
| `public static final int` | `GT1` | `26` |
| `public static final int` | `IDENTIFIER` | `53` |
| `public static final int` | `ILLEGAL_CHARACTER` | `58` |
| `public static final int` | `IMPL_OBJ_START` | `55` |
| `public static final int` | `IN_EXPRESSION` | `1` |
| `public static final int` | `INSTANCEOF` | `44` |
| `public static final int` | `INTEGER_LITERAL` | `9` |
| `public static final int` | `LBRACK` | `21` |
| `public static final int` | `LE0` | `31` |
| `public static final int` | `LE1` | `32` |
| `public static final int` | `LETTER` | `56` |
| `public static final int` | `LITERAL_EXPRESSION` | `1` |
| `public static final int` | `LPAREN` | `19` |
| `public static final int` | `LT0` | `27` |
| `public static final int` | `LT1` | `28` |
| `public static final int` | `MINUS` | `47` |
| `public static final int` | `MOD0` | `51` |
| `public static final int` | `MOD1` | `52` |
| `public static final int` | `MULT` | `45` |
| `public static final int` | `NE0` | `35` |
| `public static final int` | `NE1` | `36` |
| `public static final int` | `NOT0` | `37` |
| `public static final int` | `NOT1` | `38` |
| `public static final int` | `NULL` | `16` |
| `public static final int` | `OR0` | `41` |
| `public static final int` | `OR1` | `42` |
| `public static final int` | `PLUS` | `46` |
| `public static final int` | `QUESTIONMARK` | `48` |
| `public static final int` | `RBRACK` | `22` |
| `public static final int` | `RPAREN` | `20` |
| `public static final int` | `START_DEFERRED_EXPRESSION` | `3` |
| `public static final int` | `START_DYNAMIC_EXPRESSION` | `2` |
| `public static final int` | `STRING_LITERAL` | `12` |
| `public static final int` | `TRUE` | `14` |

| org.jdesktop.el.impl.parser.ELParserTreeConstants | | |
| --- | --- | --- |
| `public static final int` | `JJTAND` | `7` |
| `public static final int` | `JJTBRACKETSUFFIX` | `24` |
| `public static final int` | `JJTCHOICE` | `5` |
| `public static final int` | `JJTCOMPOSITEEXPRESSION` | `0` |
| `public static final int` | `JJTDEFERREDEXPRESSION` | `2` |
| `public static final int` | `JJTDIV` | `17` |
| `public static final int` | `JJTDOTSUFFIX` | `23` |
| `public static final int` | `JJTDYNAMICEXPRESSION` | `3` |
| `public static final int` | `JJTEMPTY` | `21` |
| `public static final int` | `JJTEQUAL` | `8` |
| `public static final int` | `JJTFALSE` | `28` |
| `public static final int` | `JJTFLOATINGPOINT` | `29` |
| `public static final int` | `JJTFUNCTION` | `26` |
| `public static final int` | `JJTGREATERTHAN` | `11` |
| `public static final int` | `JJTGREATERTHANEQUAL` | `13` |
| `public static final int` | `JJTIDENTIFIER` | `25` |
| `public static final int` | `JJTINTEGER` | `30` |
| `public static final int` | `JJTLESSTHAN` | `10` |
| `public static final int` | `JJTLESSTHANEQUAL` | `12` |
| `public static final int` | `JJTLITERALEXPRESSION` | `1` |
| `public static final int` | `JJTMINUS` | `15` |
| `public static final int` | `JJTMOD` | `18` |
| `public static final int` | `JJTMULT` | `16` |
| `public static final int` | `JJTNEGATIVE` | `19` |
| `public static final int` | `JJTNOT` | `20` |
| `public static final int` | `JJTNOTEQUAL` | `9` |
| `public static final int` | `JJTNULL` | `32` |
| `public static final int` | `JJTOR` | `6` |
| `public static final int` | `JJTPLUS` | `14` |
| `public static final int` | `JJTSTRING` | `31` |
| `public static final int` | `JJTTRUE` | `27` |
| `public static final int` | `JJTVALUE` | `22` |
| `public static final int` | `JJTVOID` | `4` |

| org.jdesktop.el.impl.parser.SimpleCharStream | | |
| --- | --- | --- |
| `public static final boolean` | `staticFlag` | `false` |

---


|  |  |  |
| --- | --- | --- |
| **Overview** \| Package \| Class \| Use \| **Tree** \| **Deprecated** \| **Index** \| **Help** | |  |
| PREV   NEXT | **FRAMES**    **NO FRAMES**     **All Classes** |  |


---
